# Relationship between classic indicators of health behaviour and contraceptive choices in women in Flanders

**DOI:** 10.1186/s12905-024-03079-y

**Published:** 2024-05-05

**Authors:** Nina Van Eekert, Naomi Biegel, Leen De Kort, Veronique Verhoeven, Thies Gehrmann, Caroline Masquillier, Sarah Ahannach, Sarah Lebeer

**Affiliations:** 1 Research Foundation Flanders (FWO), Brussels, Belgium; 2 Centre for Population, Family & Health, Research Foundation Flanders (FWO), Antwerp, Belgium; 3 Department of Bioscience Engineering, Research Group Environmental Ecology and Applied Microbiology, University of Antwerp, Antwerp, Belgium; 4 Department of Family Medicine and Population Health (FAMPOP), University of Antwerp, Antwerp, Belgium

**Keywords:** Contraceptive use, Local hormonal contraceptives, Hormone free contraceptives, Educational gradient, Health behaviour, Fundamental cause theory, Social diffusion of innovation theory, Naturalization, Demedicalisation

## Abstract

**Background:**

In this study we shed light on ongoing trends in contraceptive use in Flanders (Belgium). Building on the fundamental cause theory and social diffusion of innovation theory, we examine socio-economic gradients in contraceptive use and the relationship to health behaviours.

**Methods:**

Using the unique and recently collected (2020) ISALA data, we used multinomial logistic regression to model the uptake of contraceptives and its association to educational level and health behaviour (N:4316 women).

**Results:**

Higher educated women, and women with a healthy lifestyle especially, tend to use non-hormonal contraceptives or perceived lower-dosage hormonal contraceptives that are still trustworthy from a medical point of view. Moreover, we identified a potentially vulnerable group in terms of health as our results indicate that women who do not engage in preventive health behaviours are more likely to use no, or no modern, contraceptive method.

**Discussion:**

The fact that higher educated women and women with a healthy lifestyle are less likely to use hormonal contraceptive methods is in line with patient empowerment, as women no longer necessarily follow recommendations by healthcare professionals, and there is a growing demand for naturalness in Western societies.

**Conclusion:**

The results of this study can therefore be used to inform policy makers and reproductive healthcare professionals, since up-to-date understanding of women’s contraceptive choices is clearly needed in order to develop effective strategies to prevent sexually transmitted infections and unplanned pregnancies, and in which women can take control over their sexuality and fertility in a comfortable and pleasurable way.

## Background

It has so far been well established that contraceptive use is positively related to socioeconomic status (SES) and other health promoting behaviour. The fundamental cause theory of Link & Phelan (2010, 2005, 1995) describes how SES influences a person’s access to information, goods and services. This results in health disparities between people with more or fewer resources, but also in the health-promoting behaviour they display. Empirically, these associations also exist between SES and sexual and reproductive health (SRH) behaviour. European research has found that women with lower SES use less or less-reliable contraceptives for various reasons. These include having limited knowledge about SRH and contraceptives; experiencing additional barriers, such as high costs in accessing contraceptives; and contraceptive use not being the norm in their social environment [1–4]. Overall, people with a higher educational level are more likely to use contraceptives, and particularly modern methods of contraception (defined as ‘a product or medical procedure that interferes with reproduction from acts of sexual intercourse’) [5–9].

In line with fundamental cause theory, past research also shows a positive association between health promoting behaviours and contraceptive use. Adolescents with a greater orientation towards health display more regular contraceptive use, also when controlled for other socio-demographic characteristics, family composition, and pregnancy experience [10]. In contrast, health-risk behaviours, such as regular smoking, binge-drinking and drug-use, were associated with a higher chance of not using a condom [11]. Furthermore, a study in adults found that the women who used oral contraceptives were also those who took preventive measures such as cervical smear testing [12]. Using contraceptives thus seems indicative of general awareness about SRH and vice versa.

However, it seems that people’s opinion about desirable contraceptive strategies are changing. While oral hormonal contraceptives (mainly ‘the pill’) are still the predominant strategy in many countries, including Belgium [13] where the current study is situated, women are also increasingly questioning the use of hormonal contraceptives [13–17]. More women are considering replacing ‘the pill’ by alternatives modern contraceptives with either a lower hormonal dose or without hormones (e.g., long-acting contraceptive methods such as a hormonal intrauterine device (IUD) or a copper IUD, respectively), or abandoning (modern) contraceptive use altogether [16]. Overall, the trend towards abandoning hormonal or contraceptive use altogether is particularly visible in advanced economies and within the group of highly educated women [13, 14, 17–21]. This may result in a possible contradiction to the original argument that the relationship between SES and health behaviour on the one hand, and SES and contraceptive use on the other, are positively associated. This will certainly be the case if women with a higher SES abandon reliable methods of hormonal contraceptives, but the arguments could still be compatible if they switch to different non-hormonal methods.

The diffusion of innovation theory links a higher SES to an earlier onset and/or more rapid uptake of new innovations [22]. Initially the diffusion of oral contraceptive use was also positively associated with a higher SES [23]. Women first needed to have the right resources to obtain information and access to this new, innovative method. Over the past decades, information on oral contraceptives has become widespread, while access to oral contraceptives has increased through wider availability, lower prices, and better reimbursement. Having the right resources became less and less of an issue as oral contraceptives use became more normalised. Consequently, differences in use between people with a higher or lower educational level declined or disappeared. In 1997, ‘the pill’ was the most common contraceptive used in Belgium and there were no longer differences according to SES [13]. However, previous research indicates new trends are arising in contraceptive use, and consequently we expect a new SES gradient to unfold. Here, we build on the fundamental cause and diffusion of innovation theory and argue that it is important to further explore and unravel ongoing trends in contraceptive use according to SES background. This study therefore aims to investigate the association between SES, health behaviour and detailed contraceptive choices in Flanders, Belgium using recently collected data within the framework of a large-scale citizen science project, named Isala (2020; https://isala.be/en/) [24].

## Methods

### Data

In the current study we used the first wave of the citizen-science Isala study (http://www.isala.be/en/). In 2020 a survey was taken among 4.682 women between the ages of 18 and 98 years, collecting detailed information on their physical and mental health, including SRH behaviour. For the present analyses, we excluded women who self-reported to be infertile, in their menopause or older than 55 years, since we aimed to focus on women making a conscious choice about their contraceptive use. Since women might have other reasons to use contraceptives than pregnancy prevention, both heterosexual and non-heterosexual women were included in the final sample. Moreover, we only included women using reversible contraceptives and excluded those who are, or have a partner who is, sterilised. Women who were pregnant or trying to get pregnant and those who gave unclear or missing information on their contraceptive use were also excluded from the analyses. The selected sample included 4.316 women.

### Measures

#### Contraceptive use

The first (non) hormonal contraceptive variable, distinguished between using (i) hormonal methods^1^,^2^,^3^ (ii) modern non-hormonal methods (barrier^4^ and non-hormonal long-acting reversible contraceptives^5^ (hereafter LARC) and (iii) natural or no methods^6^. Here ‘hormonal’ refers to the contraceptive method including hormones, ‘modern’ refers to a product or medical procedure that interferes with reproduction from acts of sexual intercourse and ‘natural’ refers to contraceptive methods were no product or medical procedure interferes with reproduction from acts of sexual intercourse. The second dependent variable referred to contraceptive type and differentiated between: (i) oral hormonal contraceptive use^1^ (ii) non-oral hormonal short-acting reversible contraceptives^2^ (iii) hormonal LARC^3^ (iv) non-hormonal LARC^5^ (v) barrier methods^4^, and lastly (vi) natural or no methods^6^. Respondents who indicated using multiple contraceptives were categorised within the category of the most reliable contraceptive method regarding pregnancy prevention [25]. Women indicating the use of emergency contraceptives (the morning-after pill) were considered as using no method, as it may be indicative of inconsistent contraceptive use. This categorisation is in line with the Belgian Health Survey [26].

#### SES

In this analysis, educational level was used as a proxy for SES^7^, distinguishing between: (i) primary and secondary, (ii) bachelor’s degree and (iii) master’s degree and/or PhD. Due to the sampling method, a citizen-science approach method, an overrepresentation of highly educated women occurred, resulting in very few women with only primary education. We therefore decided to include them with those with secondary education.

#### Health behaviour

As indicators for health behaviour, a number of ‘healthy lifestyle indicators’, as well as ‘preventive health behaviours’ was used. Healthy lifestyle indicators were in line with the Belgian National Health Council guidelines, and activity level, diet, smoking, alcohol intake and drug use were included in the analyses. Activity level differentiated between: i) no sports ii) moderate activity (less than the recommended 150 minutes per week), iii) sufficiently active (150 minutes activity or more per week) and iv) unknown intensity. Diet differentiated between 3 categories: i) no dietary restrictions, ii) vegetarian or pescatarian and iii) vegan. Smoking differentiated between: i) non- or ex-smoker and ii) smoker. Alcohol intake included the categories i) daily and ii) less than daily, based on problematic and non-problematic drinking behaviour according to the National Health Council. Lastly drug use was categorised into, i) non- or ex-user and ii) drug user.

‘Preventive health behaviour’ consisted of three variables, two related to sexual reproductive health and one to general health, all highly encouraged and reimbursed: Human Papillomavirus (HPV) vaccination status, cervical smear and dentist. The first was HPV vaccination, where we distinguished between (i) vaccinated, (ii) not vaccinated and (iii) not known. The second indicator was whether the respondent had ever had a cervical smear, (i) yes, (ii) no and (iii) not known. Lastly, a yearly visit to dentist is recommended and actively encouraged by health insurance organisations and we included whether the respondent had been to the dentist in the past 12 months, differentiating between (i) yes and (ii) no.

#### Covariates

A number of covariates were also included in the analyses. Age was included as a continuous variable. Relationship status distinguished between women having (i) a steady partner, (ii) single women and ii) women with multiple/changing partners. Moreover, we included whether the respondent has, (i) no or (ii) one or more child(ren). Migration background, distinguishing between (i) individuals without a migration background, hereafter referred to as natives (ii) 2nd generation European Union (EU) (iii) 2nd generation non-EU (iv) 1st generation EU and (v) 1st generation non-EU. The first generation was born in the country of origin, whereas the second generation was born in Belgium but had at least one foreign-born parent. In determining background, we gave preference to the country or origin of the mother when it did not correspond to that of the father.

The descriptive statistics of all measures can be found in Table 1.

**Table 1:** Descriptive statistics of women included in the research sample (*n* = 4316)

	Range	Mean %	SD/N
**CONTRACEPTIVE USE**			
**(Non) Hormonal Contraceptive Use**			
Hormonal methods		64.85%	2799
Modern non-hormonal methods		14.94%	645
Natural or no methods		20.20%	872
**Contraceptive type**			
Oral hormonal contraceptive use		34.29%	1480
Non-oral hormonal short-acting reversible methods		8.48%	366
Hormonal long-acting reversible contraceptives		22.08%	953
Non-hormonal long-acting reversible contraceptives		3.66%	158
Barrier methods		11.28%	487
Natural or no methods		20.20%	872
**Socioeconomic status**			
**Education**			
Primary or Secondary education		22.89%	988
Bachelor		30.56%	1319
Master and/or Phd		43.56%	1880
Other		2.99%	129
**HEALTH BEHAVIOUR**			
**HEALTHY LIFESTYLE INDICATORS**			
**Activity level**			
No sports		17.38%	770
Moderate activity		21.13%	912
Sufficient activity		53%	2306
Unknown intensity		8.06%	348
**Diet**			
No dietary restrictions		84.34%	3640
Vegetarian or Pescetarian		10.10%	436
Vegan		5.56%	240
**Not Smoking**			
Non or ex-smoker		90.94%	3925
Smoker		9.06%	391
**Alcohol intake**			
Drinks alcohol less than daily		97%	4206
Drinks alcohol daily		3%	110
**Drug use**			
Non or ex-user		90.63%	3900
Drug user		9.37%	416
**PREVENTIVE HEALTH BEHAVIOUR**			
**Human Papillomavirus vaccination**			
Vaccinated		40.8%	1747
Not Vaccinated		34.24%	1478
Not known		25.28%	1091
**Cervical smear**			
Ever had a cervical smear		84.57%	3650
Never had a cervical smear		14.87%	642
Not known		0.56%	24
**Yearly visit to dentist**			
Yes		79.43%	3428
No		20,57%	888
**Constant**			
**COVARIATES**			
**Age**	18–55	30.24	7,58 SD
**Relationship status**			
Has a steady partner		75.35%	3252
Has multiple partners		2.46%	106
Single		22.20%	958
**Children**			
No children		64.83%	2798
One or more children		35.17%	1518
**Migration background**			
No migration background		86.47%	3732
1st generation EU		4.45%	192
2nd generation EU		4.68%	202
1st generation non-EU		1.85%	80
2nd generation non-EU		2.55%	110

*SD = standard deviation, N = number of observations

### Belgian context

In Belgium, most women will have to consult a doctor when they decide to take contraceptives, as any method involves a prescription or intervention [27]. Generally, women under the age of 25 can receive substantial reimbursement or free contraceptives. Certain oral hormonal contraceptives (depending on the brand) and non-hormonal IUDs are fully reimbursed, and hormonal IUDs are substantially cheaper (around 12 euro/year). For women over 25, prices can range from 10 euros per year for a non-hormonal IUD, to 140 for certain pills and 160 euros per year for certain vaginal rings, again depending on the brand.

HPV vaccination became available in 2007 and has been part of the free vaccination programme since 2010. Currently, it is offered to all children in their first year of secondary education through health check-ups organised in secondary schools [28]. Moreover, every three years women aged 24 to 64 are invited for a cervical smear, which is free of charge except for the medical consultation fee (4 euros after reimbursement) [29]. Furthermore, stimulation of dental health promoting behaviour is done by encouraging all Belgian residents to go to the dentist on an annual basis and making the reimbursement dependent on not skipping the yearly appointment [30].

## Methods

The first step was to map the contraceptive use of women included in the sample. Then, using multinomial logistic modelling, we first explored the relationship between educational level, used as a proxy for SES, and contraceptive use. Next, we included health behaviour measures, including healthy lifestyle indicators and preventive health behaviours. All models were controlled for the covariates discussed above.

Regarding the dependent variable, we first used the (non) hormonal contraceptives variable as a dependent variable and contrasted the use of (i) modern non-hormonal methods and (ii) the use of natural or no methods to (iii) hormonal methods. Secondly, the more extensive variable, contraceptive type, was used as the dependent variable. Here, the different options for contraceptive use were contrasted against the largest option which was (i) oral hormonal contraceptives; the other categories were (ii) non-oral hormonal short-acting reversible contraceptives (iii) hormonal LARC (iv) non-hormonal LARC (v) barrier, and (vi) natural or no methods.

Stata MP 17 was used to conduct all analyses.

## Results

### SES and contraceptive use

As presented in Tables 2 and 3, there were some significant differences in the use of modern non-hormonal contraceptives versus hormonal contraceptives by educational background. Compared to women who obtained only primary or secondary education, women with a bachelor’s degree were almost 1.7 times (*p* < 0.001) more likely to use modern non-hormonal methods, and women with a master’s degree or PhD were 2 times (*p* < 0.001) more likely to use modern non-hormonal methods as opposed to hormonal methods. There were no significant differences in the use of natural or no methods by educational background.

**Table 2:** Multinomial logistic regression: the association between socioeconomic position, health behavior and (non) hormonal contraceptive use

	Modern Non-Hormonal vs. Hormonal methods					Natural or no methods vs. Hormonal methods			
	RRR	P>|z|	CI			RRR	P>|z|	CI	
**Socioeconomic status**									
**Education** (ref. Primary and Secondary)									
Bachelor	1.66	0.000	1.25–2.21			1.01	0.961	0.81–1.26	
Master, PhD	2.00	0.000	1.52–2.63			1.10	0.397	0.88–1.37	
Other	1.33	0.336	0.74–2.37			0.95	0.824	0.58–1.54	
**HEALTH BEHAVIOUR**									
**HEALTHY LIFESTYLE INDICATORS**									
**Activity level** (ref. No Sport)									
Moderate	1.31	0.074	0.97–1.77			0.88	0.346	0.68–1.14	
Sufficient	1.25	0.097	0.96–1.64			0.92	0.469	0.74–1.15	
Unknown intensity	1.25	0.282	0.83–1.87			1.35	0.061	0.99–1.84	
**Diet** (ref. None)									
Vegetarian & Pesc.	2.44	0.000	1.88–3.15			1.32	0.054	1.00-1.74	
Vegan	1.25	0.260	0.85–1.83			1.25	0.205	0.89–1.75	
**Smoking** (ref. Non-smoker)									
Smoker	0.87	0.413	0.61–1.22			1.06	0.701	0.79–1.41	
**Alcohol** (ref. Daily)									
Less than daily	0.82	0.453	0.48–1.39			1.43	0.160	0.87–2.35	
**Drug use** (ref. No)									
Yes	1.32	0.072	0.98–1.79			0.93	0.625	0.68–1.26	
**PREVENTIVE HEALTH BEHAVIOUR**									
**Human Papillomavirus vaccination** (ref. Vaccinated)									
Not Vaccinated	1.43	0.002	1.14–1.81			1.41	0.002	1.13–1.74	
Not known	1.10	0.422	0.87–1.41			1.18	0.142	0.95–1.47	
**Cervical smear** (ref. Yes)									
Never	0.51	0.000	0.37–0.72			1.65	0.000	1.28–2.12	
Not known	0.20	0.128	0.03–1.58			0.96	0.951	0.29–3.17	
**Dentist** (ref. Yes)									
No	1.25	0.040	1.01–1.55			1.12	0.283	0.91–1.37	
**Constant**	0.15	0.000	0.07–0.33			0.01	0.000	0.01–0.03	

RRR = relative risk reduction, P>|z|= probability of z-statistics, CI = 95% confidence interval

source: Isala survey, Flanders Belgium, calculations by authors. (*n* = 4316)

Note: All models are controlled for age, having children, migration background, and relationship status

**Table 3:** Multinomial logistic regression: the association between socioeconomic position, health behavior and type of contraceptive use

	Non-oral short acting hormonal vs. oral short acting hormonal contraceptives				Hormonal long-acting reversible contraceptives vs. oral short acting hormonal contraceptives				Non hormonal long-acting reversible contraceptives vs. oral short acting hormonal contraceptives				Barrier vs. oral short acting hormonal contraceptives				Natural or no methods vs. oral short acting hormonal contraceptives		
	RRR	P>|z|	CI		RRR	P>|z|	CI		RRR	P>|z|	CI		RRR	P>|z|	CI		RRR	P>|z|	CI
**SOCIOECONOMIC STATUS**																			
**Education** (ref. Primary and Secondary)																			
Bachelor	1.20	0.27	0.86–1.68		1.03	0.83	0.80–1.31		1.57	0.11	0.90–2.74		1.72	0.00	1.23–2.42		1.02	0.89	0.79–1.31
Master, Phd	1.08	0.65	0.78–1.49		1.09	0.50	0.85–1.38		2.01	0.01	1.17–3.45		2.05	0.00	1.48–2.84		1.12	0.36	0.88–1.43
Other	1.66	0.11	0.89–3.10		0.82	0.49	0.47–1.43		2.62	0.04	1.06–6.50		0.91	0.82	0.42–2.01		0.93	0.81	0.53–1.63
**HEALTH BEHAVIOUR**																			
**HEALTHY LIFESTYLE INDICATORS**																			
**Activity level** (ref. No Sport)																			
Moderate	1.01	0.98	0.69–1.47		0.96	0.76	0.72–1.27		1.85	0.06	0.98–3.48		1.19	0.34	0.83–1.70		0.88	0.40	0.66–1.18
Sufficient	0.98	0.88	0.70–1.36		1.09	0.52	0.85–1.39		1.79	0.05	1.00-3.20		1.19	0.27	0.87–1.64		0.96	0.75	0.74–1.24
Unknown intensity	1.00	0.99	0.59–1.68		1.05	0.79	0.72–1.54		2.78	0.01	1.32–5.83		0.97	0.90	0.58–1.62		1.40	0.07	0.97–2.03
**Diet** (ref. None)																			
Vegetarian & Pesc.	1.09	0.70	0.71–1.68		1.82	0.00	1.34–2.48		4.15	0.00	2.63–6.56		2.76	0.00	1.99–3.84		1.66	0.00	1.21–2.27
Vegan	0.90	0.71	0.51–1.58		1.49	0.04	1.02–2.19		1.40	0.37	0.67–2.94		1.47	0.11	0.92–2.35		1.46	0.05	0.99–2.16
**Smoking** (ref. Non-smoker)																			
Smoker	0.92	0.71	0.60–1.42		1.25	0.17	0.91–1.70		1.00	0.99	0.54–1.82		0.91	0.67	0.59–1.40		1.14	0.43	0.82–1.59
**Alcohol** (ref. Daily)																			
Less than daily	0.76	0.53	0.33–1.77		0.65	0.16	0.36–1.19		0.51	0.15	0.20–1.27		0.68	0.31	0.32–1.43		1.06	0.86	0.56–2.01
**Drug use** (ref. No)																			
Yes	1.74	0.01	1.18–2.56		1.76	0.00	1.30–2.40		3.09	0.00	1.87–5.09		1.37	0.12	0.93–2.03		1.23	0.23	0.88–1.73
**PREVENTIVE HEALTH** **BEHAVIOUR**																			
**HPV** (ref. Vaccinated)																			
Not Vaccinated	0.96	0.82	0.70–1.32		1.25	0.06	0.99–1.58		1.01	0.95	0.65–1.59		1.79	0.00	1.35–2.37		1.51	0.00	1.18–1.92
Not known	0.76	0.07	0.56–1.02		0.94	0.60	0.75–1.18		1.00	0.99	0.64–1.58		1.03	0.82	0.77–1.38		1.09	0.47	0.86–1.39
**Cervical smear** (ref. Yes)	0.65	0.02	0.46–0.93		0.36	0.00	0.26–0.51		0.21	0.00	0.09–0.50		0.49	0.00	0.34–0.72		1.38	0.02	1.06–1.80
Never	2.13	0.23	0.62–7.35		2.10	0.17	0.73–6.01		0.00	0.99	0.00-.		0.45	0.46	0.05–3.79		1.47	0.56	0.40–5.49
Not known																			
**Dentist** (ref. Yes)																			
No	1.36	0.03	1.04–1.80		1.11	0.37	0.89–1.38		1.24	0.32	0.82–1.87		1.40	0.01	1.08–1.81		1.21	0.10	0.96–1.52
**Constant**	0.18	0.00	0.06–0.56		0.16	0.00	0.07–0.35		0.02	0.00	0.00-0.07		0.15	0.00	0.05–0.44		0.01	0.00	0.01–0.03

RRR = relative risk reduction, P>|z|= probability of z-statistics, CI.= 95% confidence interval

source: Isala survey, Flanders Belgium, calculations by authors (*n* = 4 316)

Note: All models are controlled for age, having children, migration background, and relationship status

Looking more specifically at the association between education and contraceptive type, we observed that the educational background of women significantly affected the use of non-hormonal LARCs over oral short-acting hormonal methods, as well as the use of barrier methods. Women with a master’s degree or PhD were twice as likely (*p* = 0.011) to use non-hormonal LARCs and 2.1 times as likely to use barrier methods as opposed to oral short-acting hormonal contraceptives, compared to women with only primary or secondary education. For women with an unknown educational level, we also observed a moderately significant higher use of non-hormonal LARCs as opposed to oral short-acting hormonal contraceptives (rrr = 2.62;*p* < 0.050) compared to women with primary or secondary education.

### Health behaviour and contraceptive use

To examine the association between contraceptive use and health behaviour, we studied healthy lifestyle indicators and preventive health behaviours (see Tables 2 and 3).

#### Healthy lifestyle

The set of healthy lifestyle variables included activity level, diet, smoking, alcohol intake and drug use. Activity level was not significantly associated with the use of modern non-hormonal contraceptives and natural or no methods compared to hormonal contraceptives. However, examining the particular contraceptive type in more depth, women with sufficient activity levels were 1.8 times more likely to rely on non-hormonal LARCs (*p* < 0.050), and those with an unknown activity level were 2.8 times more likely to use non-hormonal LARCs as opposed to oral contraceptives.

Diet was also associated with different contraceptive choices. Women who followed a vegetarian diet were more likely to use modern non-hormonal contraceptives compared to hormonal methods. More specifically, vegetarians/pescatarians were more likely to use non-hormonal LARCs (4.15 times more, *p* < 0.001), barrier methods (2.76 times more; *p* < 0.001), hormonal LARCs (1.82 times more; *p* < 0.001) and natural or no methods (1.66 times more; *p* < 0.010) compared to oral contraceptives. Similarly, vegans were 1.5 times more likely to use hormonal LARCs (*p* < 0.050) compared to women with no dietary restrictions.

There was no significant association between (non) hormonal contraceptive use and alcohol intake, smoking behaviour or drug use. When focussing on the different contraceptive types, most associations remained insignificant. However, women who engaged in drug use were significantly more likely to use non-oral short-acting hormonal methods (rrr = 1.74; *p* < 0.010) as well as hormonal LARCs (rrr = 1.76; *p* < 0.001) and non-hormonal LARCs (rrr = 3.09; *p* < 0.001) as opposed to short-acting hormonal contraceptive methods such as the pill.

#### Preventive health behaviour

Lastly, we investigated the variables indicating preventive health behaviour, such as HPV vaccination status, ever having had a cervical smear and the yearly visit to the dentist. HPV vaccination was significantly associated with hormonal contraceptive use. Women who were not vaccinated against HPV were both 1.4 times (*p* < 0.010) more likely to use modern non-hormonal methods and 1.4 times (*p* < 0.010) more likely to use natural or no methods as opposed to hormonal methods. In addition, women who were not vaccinated against HPV were more likely to use barrier methods (rrr = 1.8; *p* < 0.001) as well as natural or no methods (rrr = 1.5;*p* < 0.001) as opposed to short-acting hormonal contraceptive methods.

Furthermore, women who had never had a cervical smear were much less likely (rrr = 0.51p < 0.001) to use modern non-hormonal methods, but much more likely to use natural or no methods (rrr = 1.65;*p* < 0.001) as opposed to hormonal methods. Moreover, they were significantly less likely to adopt non-oral short-acting hormonal methods (rrr = 0.64;*p* < 0.050), hormonal LARCs (rrr = 0.36;*p* < 0.001), non-hormonal LARCs (rrr = 0.21;*p* < 0.001) as well as barrier methods (rrr = 0.49;*p* < 0.001) and more inclined to use natural or no methods (rrr = 1.38;*p* < 0.050) as opposed to oral short-acting hormonal contraceptives.

Women who had not been to the dentist in the past 12 months were moderately more likely to use modern non-hormonal methods as opposed to hormonal methods (rrr = 1.25;*p* < 0.050). In addition, they were 1.36 times more likely to use non-oral short-acting hormonal methods (*p* < 0.050) and 1.4 times more likely to use barrier methods as opposed to oral contraceptives (*p* < 0.011).

## Discussion

The current study broadens the understanding of contraceptive needs of women today. This study particularly focussed on the association between SES as well as health behaviour and contraceptive choices.

With respect to the association between contraceptive use and SES, we derived two main findings. Firstly, higher educational level, which is used as a proxy for SES, is associated with a lower use of hormonal contraceptive methods. Women with a master’s degree or PhD, followed by women with a bachelor’s degree, were most likely to use modern non-hormonal methods, especially non-hormonal LARCs and barrier methods, compared to women who only obtained primary or secondary education. The second noteworthy result was that no significant educational gradient was found for women opting for natural or no methods. This is in contrast with fundamental cause theory [31–33], stating that lower educated women experience more barriers in accessing reliable methods. We currently do not know why this is the case, but a possible explanation may be the variation within the group of women using natural or no contraception methods, as they include both women experience barriers to accessing contraceptives as ‘typical’ users of the app Natural Cycles being higher educated, financially comfortable women around the age of 30 who are in a stable/long term relationship [34–36]. This may be enforced by the selection effect of the Isala citizen-science project resulting in an over-representation of high SES women, since women were self-selected and likely already had an interest in health topics.

Regarding the association between health behaviour and contraceptive use, again we have two main findings. Firstly, our results indicated a preference for non-hormonal methods among women with a healthy, active lifestyle. Those meeting the requirements of 150 min of activity per week and following a vegetarian, pescetarian or vegan diet had a lower use of oral hormonal contraceptives in general. Compared to women with no dietary restrictions, vegetarians and vegans were more likely to use hormonal LARCs, which are often considered as lower dosage or more local hormonal contraceptives. Additionally, vegetarians were also more likely to use no or natural or no methods methods. Secondly, we discovered a group of women with a more potentially vulnerable profile: women who smoked while using hormonal contraceptives and women who did not engage in the preventive health behaviours recommended by healthcare professionals when using natural or no methods. Smoking was not associated with contraceptive use, despite the fact that hormonal contraceptives are not recommended for those who smoke due to cardiovascular risks [37]. Women who were not vaccinated were much more likely to use modern non-hormonal methods (particularly barrier) as well as natural or no methods, and those who had never had a cervical smear were less likely to use modern non-hormonal methods and more likely to use natural or no methods.

Overall, the current study clearly showed that higher educated women, and women with a healthy lifestyle especially, tended to use non-hormonal contraceptives or at least lower-dosage hormonal contraceptives that are perceived to act more locally. Over the past decade, hormonal contraceptives have increasingly been criticized by women, leading to a growing desire for more natural solutions [38, 39]. Criticism of hormonal contraceptives has included physical side effects; an (unwanted) influence on mental health; a negative impact on sexuality; concerns about future fertility; concerns about menstruation; and experiencing fears and anxiety [21, 38, 40–43]. These factors resulted in a demand for alternatives that do not interfere with the natural hormone balance of the body [21, 38, 44–46]. Rejection of hormonal contraceptives can be seen as a form of de-medicalisation as it can refer to a desire to free oneself from medical and pharmaceutical power, and thus echo the feminist “self-help” movements of the early 1970s that aimed to empower women through knowledge of their own bodies [21–23]. However, our results appear more in line with the growing demand for naturalness in Western societies [38] rather than complete de-medicalisation, since higher educated women do not fully abandon contraception but mostly opt for trustworthy alternative methods.

In line with the social diffusion of innovation theory, it is plausible that women with a higher SES have more resources to aspire to these new health values and would be the first to start doubting hormonal contraceptives and to look for alternatives appropriate to their personal needs. Moreover, in modern high-income countries, more emphasis is placed on ‘patient empowerment’ and personal control over the body (e.g., through health tracking) [48, 49]. It might be easier to obtain the required skills and knowledge for people with a higher SES and higher educational level. Women with a lower SES who generally experience more barriers in other aspects of life, often have less mental energy to dedicate to preventive care [1], including contraceptive choices. However, based on the diffusion of innovation theory we can reflect on where trends in contraceptive use are evolving. This raises the question of whether we can expect that opting for non-hormonal or natural or no methods contraceptive use is likely to further diffuse throughout the entire population.

The association between preventive health behaviour and contraceptive use is more complex and warrants more research. It is plausible that women who are not vaccinated against HPV are more likely to use modern non-hormonal methods, as well as natural or no methods, as a consequence of their desire for “healthism” and “naturalness”. In addition, their antagonism towards hormonal or unnatural methods can motivate both decisions: namely, not vaccinating and not using hormonal contraceptives. As well, it may be an indication of a lack of access to medical services, since women who had never had a cervical smear or did not go to the dentist, which are both highly recommended preventive health behaviours, had a higher inclination to use natural or no methods contraceptives. This could potentially indicate a lack of access to health services. Results in line with fundamental cause theory emphasize that women who have difficulties accessing healthcare services will also choose less reliable contraceptive methods.

A limitation of this study is the use of cross-sectional data. We suggest more research with longitudinal or qualitative data is needed to better comprehend how contraceptive use may further evolve. As well, given the need for more non-hormonal and perhaps intervention free methods, more research is needed to explore the within group variation of women using natural or no contraceptives. Different natural methods differ substantially in terms of reliability depending on how they are applied. Moreover, qualitative research on the precise contraceptive decision-making process of women in the Belgian context is necessary, preferably including information on their healthcare professionals’ advice, as they are inclined to keep on referring to hormonal methods, such as “the pill”, as their first option for contraceptives [46, 47]. Our data suggest that women do not necessarily follow recommendations by healthcare professionals and search for alternatives themselves. Learning more about how women perceive the relationship between contraceptive use and their body could help healthcare providers personalise their contraceptive counselling and customise their advice to the personal needs of women during particular times in their lives [45].

## Conclusion

Up-to-date understanding of women’s contraceptive choices is needed in order to develop effective strategies to prevent sexually transmitted infections (STIs) and unplanned pregnancies and enable women to take control over their sexuality and fertility in a comfortable and pleasurable way. Our results have implications for contraceptive use counselling: there is clearly an increasing need to offer a variety of options next to the standard contraceptive pill, allowing women to decide which is most appropriate for their needs. In addition, if natural contraceptives are still being used, such as periodic abstinence, women need to be informed about how to apply these methods in the best possible manner. This is especially necessary since the internet and social media are often sources of information, but are places where disinformation spreads easily. Healthcare professionals should be informed about these tendencies and adjust their advice accordingly, since reliable contraceptive use is still important.

## Data Availability

The datasets used during the current study are available from the corresponding author on reasonable request.

## Footnotes

1 “The pill”, pill, and minipill.

2 Hormonal anticonception ring, injection, and patch.

3 Hormonal contraceptive implant & hormonal intrauterine device (IUD).

4 Male condom, female condom (pessary, diaphragm) & spermicides.

5 Copper intrauterine device (IUD).

6 Natural methods includes coïtus interruptus, periodic abstinence, fertility awareness based method/natural family planning, lactation amenorrhea (LAM). Non-use includes using no contraceptives as well as emergency method (morning after pill; this is an emergency method and therefore not consistent contraceptive use).

7 Due to data limitations there was no possibility to include an financial income related variable. However, educational level is a widely used proxy for SES.

## Abbreviations

SES: socioeconomic status
SRH: sexual and reproductive health
IUD: intrauterine device
LARC: long-acting reversible contraceptives
HPV: Human Papillomavirus
STI: sexually transmitted infections
